# RNA biomarkers in spinal muscular atrophy: enhancing pathogenesis understanding and guiding precision medicine

**DOI:** 10.1007/s00018-026-06164-7

**Published:** 2026-03-12

**Authors:** Claudia Alberti, Angela Berardinelli, Giacomo P. Comi, Linda Ottoboni, Stefania Corti

**Affiliations:** 1 Dino Ferrari Center, Department of Pathophysiology and Transplantation (DEPT), University of Milan, 20122 Milan, Italy; 2 Casimiro Mondino Foundation, IRCCS, Pavia, Italy; 3 Neurology Complex Unit, Department of Neurosciences, Fondazione IRCCS Ca’ Granda Ospedale Maggiore Policlinico, 20122 Milan, Italy; 4 Neuromuscular and Rare Diseases Unit, Department of Neurosciences, Fondazione IRCCS Ca’ Granda Ospedale Maggiore Policlinico, 20122 Milan, Italy

**Keywords:** Spinal muscular atrophy, *SMN1*, *SMN2*, Biomarkers, Molecular profiling, RNA, RNAome, MicroRNA, Coding RNA, Non-coding RNA, Therapeutic markers, Neurodegeneration

## Abstract

Spinal muscular atrophy (SMA) is a neurodegenerative disorder resulting from mutations in *SMN1* that manifest as progressive muscle weakness and atrophy. Despite transformative therapies, such as nusinersen, risdiplam, and onasemnogene abeparvovec, heterogeneous patient responses underscore the need for reliable biomarkers to optimize treatment strategies. RNA biomarkers are particularly promising targets for monitoring SMA because the disease pathology is directly caused by impaired SMN protein, which is involved in RNA processing. Evidence suggests that SMN transcripts and specific microRNAs (i.e., miR-9, miR-206, and miR-132) are of significant diagnostic and prognostic value. In addition, specific microRNAs exhibit detectable changes in accessible biofluids at pre-symptomatic stages, enabling early non-invasive monitoring. Integration of global microRNA profiling (miRNome analysis) with clinical parameters has yielded SMA scores with high predictive value. Advancing RNA biomarker implementation requires several challenges to be addressed, including protocol standardization, validation in expanded patient cohorts, longitudinal evaluation, and seamless integration with clinical assessments. Emerging methodologies analyzing extracellular vesicle content and single-cell sequencing offer promising avenues for enhancing diagnostic precision. Multiparametric integration of RNA biomarkers may establish the foundation of precision medicine in SMA, potentially enabling individualized therapeutic selection based on molecular signatures to improve long-term patient outcomes.

## Introduction

Spinal muscular atrophy (SMA) is an autosomal recessive inherited neuromuscular disorder characterized by progressive spinal cord and brainstem motor neuron loss and severe muscle atrophy and weakness [1]. With an incidence of approximately 1 in 10,000 live births, SMA is a significant inherited cause of infant mortality [2, 3]. The disorder is caused by homozygous deletions or mutations in the survival motor neuron 1 (*SMN1*) gene that lead to a deficiency in the production of functional SMN protein, which is necessary for cellular homeostasis and viability of motor neurons [4].

The classical SMA classification delineates five phenotypic variants distinguished by age of onset and motor milestone achievement. SMA type 0 presents with profound perinatal weakness, hypotonia, and respiratory insufficiency. SMA type I (Werdnig-Hoffmann disease) manifests before 6 months of age as an inability to achieve independent sitting. SMA type II emerges between 6–18 months of age, with patients acquiring sitting but not walking abilities. SMA type III (Kugelberg-Welander disease) develops after 18 months of age, with patients initially achieving ambulation before experiencing progressive deterioration. SMA type IV presents in adulthood as mild motor deficits and preserved functional independence [5].

At a molecular level, SMN is a constitutive subunit of a multiprotein complex that is essential in small nuclear ribonucleoprotein (snRNP) biogenesis and plays a role in spliceosome assembly and pre-mRNA processing [6]. Thus, a deficiency in SMN results in widespread splicing dysregulation and disruption of axonal mRNA trafficking, ultimately resulting in compromised neuronal structure and function [7]. Disease severity inversely correlates with *SMN2* copy number. *SMN2* is a paralogous gene of *SMN1* and primarily encodes truncated and unstable SMN protein due to a critical nucleotide substitution in exon 7 that triggers its exon skipping in mRNA splicing [8]. This genotype–phenotype relationship is responsible for clinical heterogeneity in the SMA spectrum, and higher *SMN2* copy numbers are generally present in milder phenotypes [8].

Though traditionally conceptualized as a motoneuronal-restricted pathology, contemporary research has expanded our understanding of SMA as a multisystemic disorder affecting numerous tissues beyond the central nervous system. Notably, investigations have revealed muscle-specific abnormalities directly attributable to reduced SMN levels that manifest independent of neurogenic atrophy [9]. These intrinsic myopathic features include impaired myoblast fusion, aberrant myotube formation, and dysregulated muscle-specific gene expression, suggesting autonomous skeletal muscle pathology concurrent with neurodegeneration [9]. This paradigm shift necessitates a reconsideration of the fundamental pathomechanisms of SMA, with significant implications for therapeutic strategies and biomarker development.

The therapeutic landscape for SMA has undergone a revolutionary transformation with the approval of three disease-modifying therapies. Nusinersen is administered intrathecally and functions as an antisense oligonucleotide that enhances *SMN2* exon 7 inclusion, thereby increasing functional SMN protein production [10]. Risdiplam, an orally bioavailable small molecule, similarly modulates *SMN2* splicing to augment SMN expression [11]. Onasemnogene abeparvovec is administered as a single intravenous infusion and utilizes an adeno-associated viral vector to introduce functional *SMN1* cDNA [2]. These interventions have profoundly altered the natural history of SMA, particularly when initiated early, yielding unprecedented improvements in survival and motor function [2, 10, 11].

**Fig. 1:**
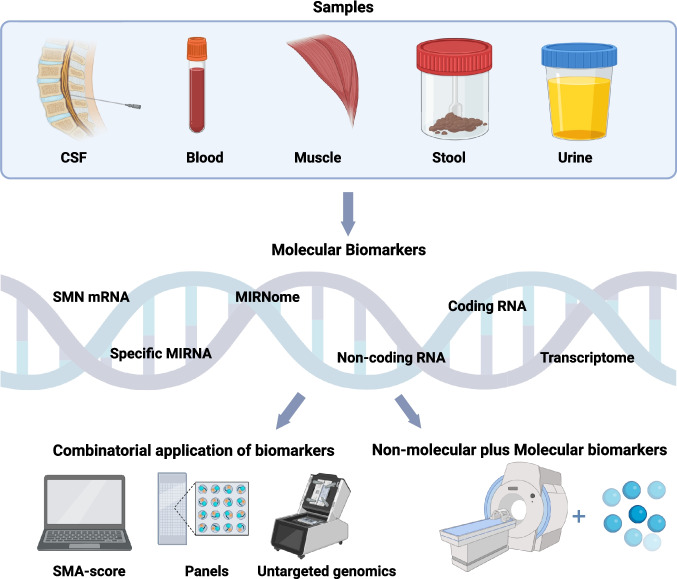
Overall scheme of the hierarchical organization of biological samples and biomarker types that have been used in spinal muscular atrophy (SMA) research and clinical monitoring. CSF, cerebrospinal fluid. The molecular biomarkers fall into several categories, including mRNA-encoding SMN, specific miRNAs, and broader analyses, such as the complete miRNome profile. Further trends are towards more comprehensive approaches, namely coding and non-coding RNA analysis, whole transcriptome studies, and combinatorial applications, including SMA scores and biomarker panels. It is this visualization that underlines the multi-faceted approach for broad biomarker analysis in SMA, highlighting the diversity in sample sources and various molecular and non-molecular targets that could be analyzed for disease monitoring, assessment of progression, and evaluation of the treatment response

Despite these therapeutic advances, significant challenges persist, including heterogeneous treatment responses and ongoing disease progression in a subset of patients [12, 13]. A variety of factors influence clinical outcomes, including *SMN2* copy number, the timing of interventions, and disease duration, but these factors only partially account for the observed variability [11]. Recent genomic investigations implicate additional genetic modifiers, such as *NAIP*, *GTF2H2*, and *PLS3*, in phenotypic determination [14]. Systematic reviews emphasize the necessity of comprehending biomarker dynamics over the natural history of SMA in order to properly analyze treatment-induced changes. The ongoing heterogeneity highlights the need for reliable biomarkers (Fig. 1) to predict the therapeutic response, track disease progression, and enable personalized therapeutic strategies with both SMN-dependent and SMN-independent mechanisms [10, 15, 16].

**Fig. 2:**
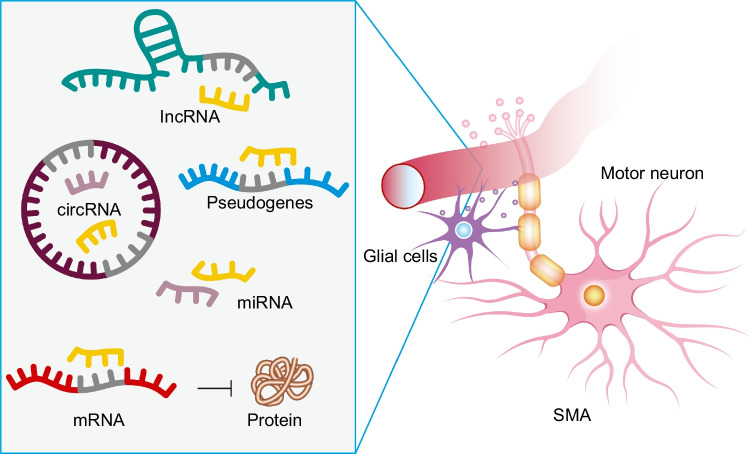
RNAs in the neuromuscular system in spinal muscular atrophy (SMA) and their interactions. A complex interplay occurs between motor neurons (blue), glial cells (purple), and various RNA species. The cellular components of the neuromuscular system affected in SMA are shown on the left. The key RNA regulatory elements and their interactions in relation to protein expression are shown on the right. lncRNA, long non-coding RNA; circRNA, circular RNA; miRNA, microRNA; mRNA, messenger RNA. Pseudogenes are also depicted, as they can generate non-coding transcripts that function as ceRNAs, contributing to the RNA regulatory landscape relevant to SMA. The non-coding RNAs participate in competing endogenous RNA mechanisms, modulating gene expression by interacting with miRNAs. The abundance and regulatory roles of the RNA species in the central nervous system make them promising candidates for non-invasive biomarkers in SMA diagnosis and monitoring because they can be detected in body fluids (inspired by [150])

As we discuss in this review, existing candidate biomarkers include the levels of SMN transcript and protein, as SMN transcript levels correlate with SMA severity and are employed as pharmacodynamic markers in SMN-targeted therapy [17], but they are hampered by temporal fluctuation and poor discriminatory power despite their pathomechanistic relevance [18]. Neurofilament light chain (NfL) is related to SMA disease activity and the response to treatment in children [19, 20], but its utility is more limited in adult patients with long illness duration [21, 22]. However, RNA biomarkers are particularly promising biomarker candidates in SMA due to the inherent defects in RNA processing [23]. The role of SMN in snRNP assembly is responsible for the pervasive changes in the transcriptome in SMA [23], and a range of RNA molecules, such as mRNAs, microRNAs (miRNAs), and long non-coding RNAs (lncRNAs) (Fig. 2), are potential candidates for biomarkers [23]. Specific miRNAs that are involved in neuron and muscle function, such as miR-9, miR-132, and miR-206, are potential candidates for non-invasive SMA monitoring [24]. LncRNAs, such as SMN-AS1, regulate SMN protein expression [25], and others, such as NEAT1 and MALAT1, regulate motor neuron viability [26] (Table 1). Furthermore, transcriptomic profiling has identified splicing defects in genes critical to motor neuron function, including *AGRN*, *CACNA1S*, and *SCNN1A*, and have been put forward as potential biomarkers of the treatment response and disease progression [27, 28].

**Table 1:** RNA Biomarkers in Spinal Muscular Atrophy (SMA)

RNA Type	Specific Marker	Sample Source	Function(s)	Key Findings	Level of Evidence	Reference(s)
mRNA	SMN2-FL (full-length)	Blood, fibroblasts, CSF	- Diagnosis: Can differentiate between healthy controls and SMA patients - Prognosis: Correlates with disease severity in SMA type I - Treatment response: Increases after risdiplam treatment	Can differentiate between SMA types; levels inversely correlate with disease severity in some studies	Moderate: Multiple clinical studies with consistent findings	Tiziano et al. [36]
mRNA	SMN-Δ7 (lacking exon 7)	Blood, fibroblasts	- Limited utility as diagnostic or prognostic biomarker - Treatment response: Reduced levels after valproic acid treatment	Expression levels similar to healthy controls; limited utility as biomarker	Low: Inconsistent findings across studies	Crawford et al. [37], Sumner et al. [42]
mRNA	*PLS3* (Plastin 3)	Blood, muscle tissue	- Prognosis: Associated with disease severity in female patients - Prediction: Indicates potential for milder phenotype in females	Associated with disease severity; increased expression linked to milder phenotypes in female patients	Moderate: Well-documented sex-specific effects	Strathmann et al., [118], Wirth [70]
miRNA	miR-9	Serum, CSF	- Diagnosis: Elevated in serum of SMA patients - Monitoring: Shows changes before tissue alterations - Treatment response: Normalizes after treatment	Upregulated in serum of SMA patients; potential for monitoring disease progression	Moderate: Replicated in multiple studies	Catapano et al. [24],
miRNA	miR-206	Serum, muscle tissue	- Diagnosis: Potential specific biomarker for SMA vs. other neuromuscular disorders - Monitoring: Dramatically increases in serum during disease progression	Upregulated in mouse models; inconsistent results in human patients	Low-moderate: Inconsistent between animal and human studies	Catapano et al. [24], Malacarne et al. [91]
miRNA	miR-132	Serum, CSF	- Diagnosis: Significantly upregulated in SMA - Treatment response: Complete normalization after treatment in animal models	Upregulated in serum of SMA patients; responds to treatment	Moderate: Consistent findings in human samples	Catapano et al. [24],
miRNA	miR-181a-5p, miR-324-5p, miR-451a	Serum	- Diagnosis: Part of composite SMA score - Prediction: Predicts phenotype in combination with other markers	Upregulated in serum in SMA; part of SMA score for predicting phenotype	Moderate: Part of validated composite score	Abiusi et al. [77]
miRNA	miR-107, miR-142-5p, miR-335-5p, miR-423-3p, miR-660-5p, miR-378a-3p, miR-23a-3p	Blood	- Prediction: Associated with treatment response - Treatment response: Predictive of response after 6 months	Associated with response to nusinersen treatment	Moderate: Prospective validation in treatment cohort	Zaharieva et al. [78]
miRNA	miR-206, miR-133a-3p	CSF	- Prediction: Lower baseline levels predict better treatment response - Treatment response: Levels decrease with therapy	Lower baseline levels in CSF predict better response to nusinersen	Moderate: Clinical correlation with outcomes	Magen et al. [79]
miRNA	miR-34 family	CSF	- Prognosis: Baseline levels predict motor skills - Treatment response: Consistent changes during treatment - Monitoring: Reflects disease progression	Downregulated during SMA progression; potential therapeutic target and biomarker	Moderate: Strong correlation with outcomes	Chen et al. [101]
miRNA	miR-23a	CSF, spinal cord, iPSC-derived motor neurons	- Diagnosis: 2.5-fold downregulation in SMA motor neurons—Prognosis: Correlates with disease severity—Therapeutic target: AAV9-mediated delivery improves outcomes in mouse models	Downregulated in SMA motor neurons; scAAV9-miR-23a delivery improves motor neuron survival, NMJ integrity, and extends survival in mouse models; neuroprotective in human iPSC-derived motor neurons	Moderate: Preclinical therapeutic validation with supportive human in vitro data	Kaifer et al. [98]
miRNA	miR-218	CSF, spinal cord	- Diagnosis: Highly expressed in motor neurons; miR-218-deficient mice develop SMA-like symptoms—Treatment response: Increased in CSF after nusinersen treatment	Essential for motor neuron identity and synaptogenesis; knockout mice exhibit severe neuromuscular failure; levels increase in CSF of SMA patients following nusinersen therapy	Moderate: Strong preclinical evidence with supportive clinical CSF data	Amin et al. [86], Welby et al. [81]
miRNA	miR-146a	CSF, iPSC-derived astrocytes	- Treatment response: Increases with treatment - Monitoring: Correlates with motor function improvements	Upregulated in astrocytes in SMA; increased expression in CSF after nusinersen treatment	Moderate: Mechanistic validation in cellular models	Welby et al. [81], Sison et al. [82]
miRNA	miR-7-5p, miR-15a-5p, miR-15b-3p/5p, miR-126-5p, miR-128–2-5p, miR-130a-3p	CSF	- Treatment response: Differential expression during treatment	Differentially expressed in CSF during nusinersen treatment	Low: Exploratory study	D’Silva et al. [76]
lncRNA	SMN-AS1	Cell lines, CNS tissue	- Diagnosis: Potential marker of SMN expression - Prognosis: May indicate disease severity	Negatively regulates SMN expression; potential biomarker for SMN expression and disease severity	Low: Limited human data	D’Ydewalle et al. [25]
lncRNA	MALAT1	Cell lines, CNS tissue	- Monitoring: Potential indicator of disease progression - Prognosis: May reflect neuronal dysfunction	Dysregulated in SMA models; potential biomarker of disease progression and neuronal dysfunction	Low: Primarily preclinical evidence	Burghes et al. [26]
circRNA	SMN circ4-2b-3	Exosomes, serum	- Monitoring: Could reflect SMN gene activity - Treatment response: Potential indicator of treatment effects	Elevated in subset of patients with superior treatment response to nusinersen	Low-moderate: Emerging evidence in clinical cohort	Guerra et al. [107]
Panel	SMA score	Serum	- Diagnosis and classification: Distinguishes between SMA types - Prognosis: Predicts disease course	Combination of miRNAs with *SMN2* copy number and patient age improves accuracy	Moderate: ~ 75% accuracy in phenotype prediction	Abiusi et al. [77]
Other	Exosomal RNAs	Plasma, serum	- Diagnosis: Disease-specific signatures - Monitoring: Enable non-invasive disease tracking	Disease-specific RNA signatures detected in extracellular vesicles from patient plasma	Low-moderate: Growing evidence base	Lin et al. [122], Sproviero et al. [138]
Other	cfRNA	CSF	- Treatment response: Differential expression after treatment - Monitoring: Reflect molecular changes during therapy	48 differentially expressed cfRNAs identified after nusinersen treatment	Low: Exploratory study	Zhong et al. [123], Leo et al. [126]
Global	Transcriptome-wide changes	iPSC-derived motor neurons, muscle tissue, spinal cord	- Diagnosis: Comprehensive molecular signature - Therapeutic target identification	> 1,000 dysregulated genes and 12,000 altered splice events identified	Moderate: Consistent patterns across studies	Rizzo et al. [136]

*miRNA* microRNA; *circRNA* circular RNA; *lncRNA* long non-coding RNA; *cfRNA* cell-free RNA; *CNS* central nervous system; *CSF* cerebrospinal fluid; *iPSC* induced pluripotent stem cell

Despite these advances, no biomarker has yet been reliable enough to guide clinical decision-making independent of other information. Overcoming this challenge requires strict validation in large patient cohorts, longitudinal measurement, and standardized approaches to enable personalized SMA management.

## SMN transcripts as biomarkers

SMN protein expression is developmentally regulated, with prominent peaks in late fetal life and a subsequent 2.3-fold reduction after birth [1, 29]. This pattern of expression in development is in synchrony with phases of motor neuron development and neuromuscular junction (NMJ) formation. The levels of SMN transcripts have considerable tissue variation, with levels being lower in blood than in motor neurons [30, 31].

SMN-encoding mRNA, including full-length *SMN1* mRNA (*SMN1*-FL), *SMN2*-FL, and SMN transcripts lacking exon 7 (SMN-Δ7), directly correspond to the primary etiological factor in SMA – insufficient SMN protein in motor neurons [32–35]. *SMN2* harbors a critical C-to-T transition within exon 7 that results in its exclusion from approximately 90% of transcripts, generating a truncated, rapidly degraded protein [36, 37].

Given their intrinsic connection to the molecular pathogenesis of SMA, these transcripts potentially provide valuable insights into the disease mechanisms, severity, and therapeutic responsiveness.

### Measurement strategies

SMN transcripts can be quantified using several molecular approaches, including real-time PCR in leukocytes [36], low-abundance transcript detection by droplet digital PCR, and testing of peripheral blood cells, fibroblasts [37, 38], and cerebrospinal fluid (CSF) [39]. More recent approaches include RNA sequencing (RNA-seq) for global splicing pattern analysis and NanoString for multiplex analysis. A 2022 comparative evaluation of three measurement approaches (real-time RT-PCR, quantitative fluorescent RT-PCR, and semiquantitative RT-PCR) represented progress toward standardized measurements [17].

Despite the demonstrated sensitivity and specificity, measurement of SMN transcripts is limited by tissue expression patterns and variations in methodology. There is no agreed-upon protocol for measuring SMN-encoding mRNAs and, thus, the development of standardized transcript-based biomarkers is limited. Kolb et al. utilized droplet digital PCR to demonstrate lower *SMN* mRNA levels in infants with SMA compared to controls [40].

### Clinical evidence and diagnostic utility

Early investigations demonstrated that levels of full-length SMN transcripts could differentiate SMA patients, particularly those with SMA type I, from controls [36, 38]. However, some studies reported no correlation between SMN-FL expression and clinical phenotype [37]. SMN-Δ7 levels in peripheral blood cells and fibroblasts were found to be comparable between SMA patients and controls [37, 38], highlighting tissue-dependent variations in splicing regulation. Comparative analyses revealed substantial overlap between patients and controls despite group differences, limiting the diagnostic utility of these transcripts in isolation [41].

Using quantitative RT-PCR, Sumner et al. demonstrated that full-length SMN transcript levels correlate with *SMN1* and *SMN2* copy numbers, whereas SMN-Δ7 levels primarily depend on *SMN2* copy number [42]. Additional research identified significant differences in SMN-FL and SMN-Δ7 levels between controls and various SMA subtypes [43].

The SMN-FL to SMN-Δ7 ratio has emerged as a potentially critical parameter for distinguishing patients from carriers and controls [44]. Quantitative fluorescence measurements revealed that full-length, SMN-Δ7, and total *SMN2* transcript levels increase linearly with *SMN2* copy number, inversely correlating with disease severity [45]. Maretina et al. reported that the proportion of SMN-FL to total SMN-encoding mRNA (SMN-FL + SMN-Δ7) effectively discriminated SMA patients, carriers, and normal individuals with greater sensitivity than the real-time PCR-based ratio of SMN-FL:SMN-Δ7 and sensitivity to therapeutic oligonucleotides in SMA fibroblasts [17].

### Monitoring the therapeutic response

Trials of SMA type II patients have demonstrated elevated SMN FL-transcript in extracellular vesicles following 14 months of nusinersen therapy, with potential implications for non-invasive monitoring [46]. The levels of full-length *SMN* transcripts and SMN protein levels were both elevated by risdiplam in infants (age 1–7 months) with SMA type I [33] and in healthy adult males (age 18–45 years) [47]. Children with SMA type II or III had variable responses to nusinersen that correlated with SMN protein elevations in the CSF [39].

In contrast, small molecule therapies have yielded inconsistent results. The SMN transcript levels served as longitudinal biomarkers in clinical trials [48]. Histone deacetylase inhibitor studies produced variable outcomes, with one phase 2 trial showing unchanged SMN-FL levels but reduced SMN-Δ7 levels following valproic acid administration [49, 50]. However, salbutamol therapy has been shown to substantially increase SMN-FL transcript levels, which are significantly reduced in leukocytes from SMA patients compared to controls and correlate with disease severity [50].

### Challenges and constraints

Technical constraints to using SMN transcripts as biomarkers include transcript instability [51], degradation of RNA during sample processing, and requirements for the precise quantification techniques. Biological constraints consist of extensive variability and overlap of SMN transcript and protein levels in SMA patients and controls [38, 42, 52, 53]. The BforSMA Clinical Study showed poor correlation of SMN protein/mRNA levels with clinical endpoints [37]. In addition, the correlation of peripheral SMN expression and expression in the central nervous system remains poorly characterized.

Analytical parameters, including method selection, normalization approaches, reference gene choices, and other variables, significantly influence results. Under optimized conditions, the levels of SMN-FL and SMN-Δ7 can serve as informative biomarkers for disease progression and therapeutic efficacy, though their predominant availability in blood rather than the primary therapeutic target, the spinal cord, represents a notable limitation.

### Future directions

As SMN transcripts effectively distinguish between SMA patients and controls, with levels correlating with clinical severity in SMA type I patients [36, 42], they can serve as pharmacodynamic biomarkers to confirm target engagement of SMN-enhancing therapies [5, 54], providing direct mechanistic evidence of therapeutic activity through transcript modulation. Future research agendas include the creation of more specific and sensitive methods for detection of SMN transcripts in clinically accessible tissues (e.g., peripheral blood cells, fibroblasts, and cerebrospinal fluid) using new single-molecule detection approaches. Other targets include RNA-binding proteins and epigenetic mechanisms controlling *SMN2* splicing [55] and multimodal approaches with more than one biomarker [39]; other biomarkers, such as NfL, will be important [56]. Single-cell RNA-seq, spatial transcriptomics, and longitudinal sampling are new technologies that have greater potential. Longitudinal prospective studies that correlate molecular markers with functional outcomes will be essential for clinical validation.

Though SMN transcripts are reasonable biomarkers because of their direct relationship with the disease mechanism, combining them with other biomarkers will most likely be essential. As SMA treatment continues to be refined, SMN transcript biomarkers will have increasingly important roles in personalized medicine approaches to treatment selection and timing.

## Circulating microRNAs: promising non-invasive biomarkers

MiRNAs are small non-coding RNAs (ncRNAs) that post-transcriptionally regulate gene expression by targeting 3'-UTR sequences, either inhibiting translation or inducing mRNA degradation. Approximately 30% of human genes undergo miRNA-mediated regulation. These regulatory molecules play critical roles in both neuron and muscle development, establishing their relevance in neurodegenerative and neuromuscular disorders. Remarkably, global miRNA dysfunction, demonstrated in Dicer-deficient mice, produces an SMA-like phenotype. Their tissue-specific expression patterns and remarkable stability in biological fluids make miRNAs excellent candidates for non-invasive disease biomarkers [57, 58].

In circulation, miRNAs maintain stability through an association with protective carriers, such as exosomes, Argonaute proteins, and high-density lipoproteins [59]. Extracellular vesicles, particularly exosomes (30–150 nm vesicles derived from the endosomal pathway), serve as crucial transporters of these disease-relevant molecules, positioning them as important targets for SMA biomarker research [60].

### Relevance to SMA

MiRNAs are particularly relevant in neuromuscular diseases because of their regulatory functions in muscle and neuron development (i.e., in the maintenance and differentiation of motor neurons) [61, 62]. In normal cellular processes, miRNAs undergo sequential processing from primary transcripts through Drosha/DGCR8 and Dicer-mediated maturation before forming the RNA-induced silencing complex [63]. In SMA, SMN deficiency disrupts these ribonucleoprotein complexes by affecting ribonucleoprotein biogenesis of messengers and directly affecting miRNA functions [64], leading to dysregulation of the miRNAs essential to motor neuron development and function [64, 65]. Distinctive miRNA expression patterns have been demonstrated in SMA model systems and patient tissues, with certain miRNAs having a direct role in etiology, including miR-9 and miR-206, which are responsible for NMJ formation and maintenance [66, 67], and miR-218, which is responsible for motor neuron identity by suppressing other neural identities [68]. Several aberrantly expressed miRNAs, such as miR-183 and miR-375, are also part of the pool of candidate biomarkers.

### Diagnostic and monitoring utility

The distinctive expression profiles of miRNAs facilitate their application in diagnosis, prognosis, and treatment monitoring. Recent studies have identified specific panels that enhance diagnostic specificity compared to the measurement of individual miRNAs, including miR-181a-5p, miR-324-5p, and miR-451a in serum from SMA patients [69].

The presence of miRNAs in readily accessible biofluids makes them promising candidates for non-invasive diagnostics and therapeutic monitoring, as they have distinctive expression profiles in different SMA types and different sensitivities to therapeutic intervention [62, 70]. MiRNAs provide additive information to traditional biomarkers, as they provide information on regulatory network dysregulation, whereas SMN transcripts reflect the underlying molecular defect and neurofilaments track neurodegeneration [56].

### Challenges and future directions

Despite their promise, the utilization of miRNAs as SMA biomarkers faces several challenges, including methodological heterogeneity in isolation, normalization, and detection [71]. Many miRNAs are not tissue-specific; therefore, rigorous validation of SMA-specific signatures is necessary [72].

Future efforts will be aimed at establishing standardized protocols and validating them in large cohorts for integration with ancillary biomarkers. Longitudinal studies of miRNA patterns during the course of disease and the response to treatment, in combination with next-generation sequencing and machine learning, will enhance their clinical utility [73].

## Whole miRNome analysis in SMA

The miRNome constitutes the entirety of miRNAs present within a particular tissue or disease state. MiRNome analysis has emerged as a comprehensive tool for profiling global miRNA expression related to disease pathogenesis. In SMA, this analysis encompasses the full spectrum of miRNAs involved in motor neuron and axonal development and function, as well as skeletal muscle formation and NMJ organization. These miRNAs serve as critical post-transcriptional regulators of gene expression and have been implicated in various neurodegenerative conditions. Recent findings suggest an active role of muscle in SMA pathogenesis, aligning with contemporary advances in neuromuscular disease research [9].

### Measurement strategies

MiRNome analysis involves a variety of intricate approaches in a large array of biological samples. Researchers have conducted large-scale miRNA profiling in muscle biopsy samples, cell culture, and a variety of biofluids, including serum, plasma, and CSF [74]. Methodological approaches typically comprise small RNA-seq for large-scale detection and subsequent RT-quantitative PCR validation of individual candidates [74].

Next-generation sequencing has enhanced miRNome analysis by enabling the detection of known and novel miRNAs with higher sensitivity at high-throughput. Various normalization methods (spike-in or housekeeping miRNAs) may have a significant effect on results and necessitate standardization [75]. Modern analysis pipelines involve specific RNA extraction, adapter ligation, miRBase alignment, and analysis of differential expression using optimized computational tools for short RNA sequences. Advanced bioinformatics pipelines with machine learning algorithms have further optimized the detection sensitivity and measurement of miRNA expression profiles.

MiRNome analysis offers a broader perspective than single miRNA analysis, identifying patterns and networks of dysregulation that provide more comprehensive insights into disease mechanisms. This systems biology approach enables the identification of key regulatory nodes within miRNA networks that may serve as potential therapeutic targets and complement other RNA biomarkers, such as SMN transcripts for assessment of molecular efficacy and circulating miRNAs for non-invasive monitoring [56].

### Relevance to SMA

MiRNAs play a critical role in SMA pathogenesis, with > 100 differentially expressed miRNAs in muscle biopsy and culture samples from SMA patients compared to controls [76]. Whole-miRNome analysis of muscle and culture samples of myoblasts and myotubes from SMA patients identified five miRNAs that are over-expressed in every sample group when compared with controls [77]: miR-1, miR-133a, miR-133b, miR-204-5p, and miR-208b. These dysregulated miRNAs regulate fundamental processes disrupted in SMA, including muscle differentiation, axon growth, and motor neuron development. Of particular importance is that four of the five miRNAs that are universally overexpressed belong to the muscle-specific family of miRNAs, myomiRNAs, that are responsible for the regulation of myogenesis and muscle atrophy [77]. Overexpression in muscle and serum makes them potential biomarkers for diagnosis and prognosis. Their roles in cellular growth, neurogenesis, and neuron differentiation and their actions through the mTOR and MAPK signaling pathways make them significant in disease mechanisms [76].

### Clinical studies

Several pivotal clinical investigations have advanced our understanding of miRNome alterations in SMA. Among the > 100 miRNAs differentially expressed in muscle samples from SMA patients compared to healthy controls, Abiusi et al. [77] identified three significantly upregulated miRNAs in serum samples: hsa-miR-181a-5p, hsa-miR-324-5p, and hsa-miR-451a. They developed an innovative SMA score that combines these miRNA findings with *SMN2* copy number, *SMN2*-FL transcript levels, and patient age, achieving approximately 75% accuracy in predicting SMA phenotype [77].

In blood samples, Zaharieva et al. [78] identified 69 dysregulated miRNAs between SMA patients and controls. Seven specific miRNAs (miR-107, miR-142-5p, miR-335-5p, miR-423-3p, miR-660-5p, miR-378a-3p, and miR-23a-3p) were associated with the therapeutic response to nusinersen at baseline, six of which (excluding miR-23a-3p) were predictive of the response after 6 months of treatment [78]. Further analysis revealed that miR-378a-3p levels specifically correlated with improved CHOP-INTEND and HINE scores [69].

Magen et al. [79] discovered that lower levels of muscle-specific miRNAs (miR-133a-3p and miR-206) in the CSF at baseline predicted a better clinical response to nusinersen treatment in SMA type II and III patients. These myomiRs, alone or in combination, correlated with treatment outcomes after 6 months of therapy, with miR-206 levels also inversely correlating with Hammersmith Functional Motor Scale Expanded (HFMSE) scores [79]. This finding aligns with previous observations of significant decreases in myomiR-133a, myomiR-133b, and miR-1 expression in serum from SMA type II and III patients after 6 months of nusinersen treatment compared to baseline [80].

Furthermore, following nusinersen treatment, Welby et al. [81] found increased levels of miRNAs previously downregulated in SMA models, which correlated with motor function improvements in patients with SMA type I and II [81]. Sison et al. [82] demonstrated that astrocyte-produced miR-146a is significantly upregulated in SMA, to levels that are sufficient to induce motor neuron loss in vitro, whereas its inhibition prevented astrocyte-induced motor neuron degeneration. D'Silva et al. [76] identified 66 miRNAs with significantly altered expression in CSF compared to pre-treatment levels in six nusinersen-treated SMA patients, with 14 miRNAs predicted to regulate genes involved in motor neuron survival and SMA pathogenesis.

Thus, miRNAs have demonstrated utility in differentiating between SMA patients and controls for diagnosis, with specific profiles associated with disease status. Certain miRNAs have also shown the ability to predict the treatment response, particularly to nusinersen therapy. In monitoring the response to treatment, multiple studies have shown that changes in miRNA levels correlate with therapeutic outcomes. The composite SMA score has shown particular value in phenotype prediction, especially for patients with three copies of *SMN2*.

### Challenges and limitations

Several limitations exist in current research into the miRNome in SMA, including relatively small sample sizes and limited follow-up periods in studies. The cross-sectional nature of some studies necessitate validation in prospective cohorts. Technical challenges, such as inconsistent normalization methods and varying RNA extraction protocols, affect reproducibility across different laboratories. The heterogeneity in miRNA expression across different biological fluids and tissues presents additional challenges in establishing reliable biomarker panels. There is also a need for validation in larger, more diverse patient populations to confirm the utility of miRNome-based biomarkers across different demographic analyses and in SMA subtypes. The complex interplay between miRNAs and their target genes, as well as the influence of genetic background and environmental factors on miRNA expression, further complicates the interpretation of miRNome data.

### Future directions

Future research in SMA at the miRNome level would focus on validation in large cohorts, normalization with the same methods, establishing age-adjusted reference ranges, combining miRNA biomarkers with clinical features through machine learning, and the application of multi-omics. Other research priorities to enable personalized treatment are to explore miRNA modulation as a therapeutic target, miRNA-based liquid biopsy for non-invasive monitoring, and miRNAs in extracellular vesicles in SMA pathogenesis.

As critical and diverse miRNAs involved in neuromuscular development are disrupted in serum, plasma, CSF, and muscle tissue in SMA patients, they are biomarkers of the disease mechanism and promising presymptomatic biomarkers and markers of the treatment response. Therefore, miRNAs are valuable in monitoring the therapeutic response and in predicting prognosis in SMA.

## Specific neuromiRs in SMA pathogenesis as biomarkers

NeuromiRs, particularly miR-9, miR-124, miR-132, and miR-218, are predominantly expressed during early neural development and exhibit crucial functions in neurogenesis while simultaneously suppressing gliogenesis. These highly conserved miRNAs demonstrate distinct neural functions across vertebrates.

### NeuromiRs of interest

MiR-9 regulates neural stem cell proliferation and differentiation by targeting specific transcription factors and influences motor neuron columnar organization through FoxP1 regulation [83, 84]. Interestingly, miR-9 exhibits tissue-specific dysregulation in SMA; it is downregulated in motor neurons and spinal cord in mouse models but upregulated in patient blood samples, potentially contributing to neurofilament-associated motor neuron degeneration [24].

MiR-124 plays essential roles in normal neuron formation, particularly in motor neurons, and contributes to regulation of synaptic structure and function, demonstrating dysregulation in SMA samples [85]. MiR-132 critically modulates neuronal dendritic outgrowth, synaptic plasticity, and neovascularization [24, 62], with reduced expression in spinal cords from SMA mouse models but increased levels in serum from SMA patients [24].

MiR-218, which is highly expressed in motor neurons, significantly contributes to synaptogenesis. Notably, miR-218-deficient mice develop severe SMA-like symptoms [86], highlighting its importance in motor neuron function. Following nusinersen treatment, miR-218 is increased in CSF samples from SMA patients compared to pre-treatment levels [81].

### Measurement strategies

The gold standard for measuring neuromiRs continues to be real-time RT-PCR, which has high sensitivity and single-nucleotide specificity [87]. Other methods with varying sensitivity are droplet digital PCR, microarrays, and RNA-seq. Technical challenges are low miRNA concentrations in biofluids; variation in normalization, making it difficult to interpret results; and variation in the RNA extraction efficiency between sample types [88]. Pre-analytical sample handling with standardized operating procedures has a significant impact on measurement reproducibility and reliability.

### Relevance to SMA

NeuromiRs are central to SMA pathogenesis because they are involved in motor neuron development, guidance, and formation of synapses. They regulate genes involved in the growth and guidance of axons to target muscles for proper connection and function [84]. MiR-9, in particular, is involved in the determination of subtypes of motor neurons, growth of dendrites, and synaptic function. Dysregulation of miR-9 can cause abnormal development of motor neurons via FOXP1 patterning and abnormal neurite growth [24, 62].

In mouse models of severe SMA type I, miR-9 exhibits tissue-specific dysregulation, with downregulation in the spinal cord and upregulation in the serum and skeletal muscle. Serum miR-9 levels increase 3.5-fold at presymptomatic stages (post-natal day 2, PND2), becoming more pronounced (4.4-fold) by mid-symptomatic stages (PND7). Similarly, miR-132 exhibits tissue-specific dysregulation in SMA models, with serum levels dramatically increasing fivefold by mid-symptomatic stages [24, 62].

Human studies have confirmed significant upregulation of miR-9 (2.36-fold) and miR-132 (1.54-fold) in serum from SMA type II patients compared to healthy controls [24], supporting their potential clinical utility as biomarkers [24].

### Function in diagnosis and monitoring

NeuromiRs have potential as diagnostic markers due to their tissue-specific dysregulation patterns and early elevation before tissue changes are evident. Their neural specificity provides insights into neuronal pathology not captured by broader miRNA panels. NeuromiRs can complement other RNA biomarkers, such as SMN transcripts for the monitoring of molecular efficacy, and allow more comprehensive miRNome profiling, providing distinctive neural insights with direct applicability to motor neuron pathology [56].

Both miR-9 and miR-132 exhibit a therapeutic response. In FVB.Cg-Tg(*SMN2*)2Hung *SMN1*tm1Hung/J mice with a severe SMA-like phenotype, serum levels of miR9 normalized following treatment with PMO25, a 25-mer therapeutic antisense oligonucleotide designed to increase *SMN2* exon 7 inclusion. MiR-132 showed complete normalization in serum, skeletal muscle, and spinal cord following treatment. This normalization was most pronounced at the mid-symptomatic time point (PND7), with miR-132 having the most consistent response in all tissues examined.

### Challenges and future directions

The clinical translation of neuromiRs as SMA biomarkers faces several obstacles. Challenges include low concentrations in biofluids, necessitating highly sensitive detection methods; tissue-specific expression patterns complicating blood-CNS correlations; inter-patient variability affecting reliability; overlapping expression with other neurological conditions, impacting specificity; and lack of standardized protocols, hindering cross-study comparisons [69].

Future studies need to validate neuromiR biomarkers in large and diverse patient cohorts and establish standardized measurement protocols. The highest priorities are to create neuromiR panels using machine learning algorithms to enhance diagnostic accuracy, integrate neuromiRs with other classes of biomarkers for comprehensive disease profiling, and conduct multicenter longitudinal trials to determine clinical utility. The unique capability of neuromiRs to non-invasively reflect neuron pathology makes them superior tools for monitoring disease progression and treatment efficacy in the management of SMA.

## MiR-206 and myomiRs: pivotal players in neuromuscular disorders and promising biomarkers for SMA

MyomiRs include miR-1, miR-133a, miR-206, miR-208a/b, miR-499, and miR-486. Among these, miR-206 has particular significance because it is exclusively expressed in skeletal muscle, predominantly within slow-twitch type I fibers. The muscle-associated miRNA network has also expanded to include "MotomiRs" (miR-9, miR-124, miR-218, and miR-375) that regulate motor neuron growth and function [67, 84]. Together, these miRNAs form a sophisticated regulatory network that is responsive to changes in muscle activity and are crucial for muscle growth, regeneration, and NMJ formation.

### MiR-206

MiR-206 targets numerous genes, including *HDAC4*, *FGFBP1*, *Pola1*, *BDNF*, *Igfbp5*, *Cx43*, *Notch3*, *Pax3/7*, *Utrn*, and *Fstl1* [24, 67]. Although miR-206-knockout mice do not display an overt phenotype under normal conditions, they exhibit delayed recovery following denervation, highlighting the critical role of miR-206 in the stress response and muscle repair [89]. Furthermore, miR-206 contributes to neuromuscular synapse regeneration and demonstrates neuroprotective effects in SMA and Amyotrophic Lateral Sclerosis (ALS) models. Its elevated levels in the serum of neuromuscular disease patients has been interpreted as a compensatory response [90] and suggest its potential as a non-invasive biomarker [24, 91]. The proposed neuroprotective mechanism operates through the HDAC4-FGFBP1 pathway following NMJ denervation, as it is induced in muscle cells to enhance endplate reinnervation and potentially modulate disease progression. SMA type I mouse models exhibit dynamic changes in serum miR-206 levels that significantly differ from controls and normalize with systemic antisense oligonucleotide therapy [24].

### Measurement and relevance

The measurement of miR-206 and other myomiRs is primarily done by real-time RT-PCR, a gold standard technique with very high sensitivity and specificity [92]. The technique is very sensitive at low concentrations with difficulties in laboratory-to-laboratory standardization.

MiR-206 is particularly notable in SMA due to its role in the response to injury and repair and regeneration [93]. Upregulation of miR-206 in response to motor neuron injury induces nerve regeneration through the regulation of genes responsible for muscle reinnervation and regeneration [67]. Upregulation of miR-206 induces a protective mechanism that enhances muscle endplate reinnervation through the HDAC4-FGFBP1 axis by suppressing HDAC4 protein and inducing FGFBP1 mRNA.

### Evidence from animal studies

Catapano et al. [24] demonstrated significant increases in miR-206 in both spinal cord and skeletal muscle from mouse models of severe SMA during late stages of disease. Using FVB.Cg-Tg(*SMN2*)2Hung *SMN1*tm1Hung/J (TJL005058) mouse models, severe SMA-like mice [(*SMN2*)2^±^; Smn^−/−^] had substantially increased miR-206 levels in both skeletal muscle and spinal cord compared to heterozygous controls [(*SMN2*)2^±^; Smn^±^]. Serum miR-206 levels increased tenfold at the mid-symptomatic stage (PND7). Similarly, moderate SMA mice [(*SMN2*)2^+/+^; Smn^−/−^] exhibited an eightfold increase in serum miR-206 at PND10 [24]. This upregulation likely represents a protective response to NMJ maturation defects in SMA, but it is insufficient to preserve motor neuron integrity.

Catapano et al. [24] also observed tissue-dependent abundance patterns, with skeletal muscle miR-206 levels exceeding spinal cord levels > 1,000-fold. In control mice, miR-206 exhibited developmental regulation, with levels declining from PND2 to PND7, followed by a 3.3-fold increase from PND7 to PND10 [24].

### Clinical studies

Catapano et al. [24] also thoroughly investigated miRNA expression in a variety of tissues in SMA patients. Interestingly, no significant miR-206 alteration was observed in serum from SMA patients vs. control serum. However, they noted upregulation of miR-9 (2.36-fold) and miR-132 (1.54-fold) in serum from SMA type II patients and intermediate values in SMA type III patients.

Bonanno et al. [94] compared four muscle-specific miRNAs (miR-1, miR-133a, miR-133b, and miR-206) in 21 children with SMA type II or III before and after 6 months of nusinersen treatment. Their analysis revealed decreases in all four myomiRs following therapy, with three (miR-133a, miR-133b, and miR-1) showing significant reductions. Notably, the reduction in miR-133a by at least 6.6 cycle threshold values was strongly associated with clinical improvement, reflected by increases of ≥ 3 points on the HFMSE [94].

Malacarne et al. [91] compared myomiR levels across three motor neuron diseases (ALS, Spinal and Bulbar Muscular Atrophy (SBMA), and SMA) and found significant miR-206 upregulation in serum samples from ALS and SMA patients, with similar trends in SBMA cases. These findings, apparently contradicting Catapano's results [24], suggest the potential of miR-206 as a non-invasive biomarker reflecting skeletal muscle pathophysiology across various neuromuscular conditions [91]. This discrepancy may reflect the inherent variability and reproducibility challenges in miRNA analysis.

### Functions of the biomarkers

MiR-206 and myomiRs are multifunctional SMA biomarkers; they exhibit diagnostic potential with distinct expression profiles compared with other neuromuscular disorders, and differential profiles between severe and mild SMA subtypes suggest prognostic value. Their value among serum miRNAs is increased by the fact that they change early, before alterations in the spinal cord and skeletal muscle tissue, and before clinical symptoms arise. In the context of monitoring the therapeutic response, myomiRs have also been helpful in following the response to nusinersen therapy, with changes in concentration mirroring clinical improvement [62]. Catapano et al. demonstrated that miR-9, miR-206, and miR-132 all respond very well to antisense oligonucleotide therapy, with the potential for use as early therapeutic response biomarkers [24].

MyomiRs complement other SMA biomarkers, including neuromiRs and SMN transcripts, in providing muscle-specific insights, forming an even more informative disease pathology profile when combined [56].

### Challenges and future directions

The utilization of myomiRs as SMA biomarkers faces several challenges, including high inter-patient variability, modest changes in the expression level, and contradictory findings between studies. Small sample sizes, limited follow-up periods, and technical standardization issues further complicate cross-study comparisons.

Future research needs to focus on the validation of miR-206 expression patterns in large well-characterized patient cohorts using standardized methods, the clarification of conflicting data using a multicenter cooperative research, and examining the therapeutic use of the modulation of miR-206 expression.

Advanced machine learning and sequencing technologies are likely to facilitate the detection of optimal panels of myomiRs with improved diagnostic specificity [95]. Multi-modal biomarkers can enhance diagnostics and treatment monitoring. Longitudinal studies on changes in miR-206 expression during disease progression and the response to treatment will be critical to determining its application in personalized SMA treatment.

## MiRNA fingerprinting of different neuromuscular conditions

Mousa et al. [96] investigated miRNAs as differential biomarkers across neuromuscular disorders, analyzing 74 patients with various conditions alongside 30 healthy controls. Their research identified disorder-specific miRNA signatures with significant diagnostic potential.

MiR-499, which indicates muscle damage, was upregulated across all conditions following a severity-aligned hierarchy: Duchenne muscular dystrophy (DMD) > limb-girdle muscular dystrophy (LGMD) > SMA > Becker muscular dystrophy (BMD) > congenital muscular dystrophies > myopathies. This pattern positions miR-499 as a valuable biomarker for distinguishing DMD from other neuromuscular conditions, as it demonstrated the highest diagnostic accuracy (area under the curve = 0.931) in receiver operating characteristic curve analyses.

For SMA, miR-206 emerged as a potential marker for diagnosis with significant dysregulation compared to other diseases, in the pattern SMA > LGMD > myopathy > BMD > congenital muscular dystrophy > DMD. A differential marker emerged in the form of reduced miR-208a levels in SMA patients. MiR-103a-3p discriminated well between DMD (highest expression) and BMD (lowest expression), and miR-223 was upregulated in muscular dystrophies but consistently dysregulated in SMA.

Malacarne et al. [91] built on this work by comparing miRNA profiles among SOD1-mutated ALS, SBMA, and SMA—three diseases with a common skeletal muscle component despite their diverse onset, course, and genetics. Analysis of myomiRs (miR-206, miR-133a, miR-133b, and miR-1) and their targets identified similar patterns of dysregulation in both a mouse model and patient serum. In particular, miR-206 was consistently upregulated in both mouse muscle and human serum samples.

Although conventional genetic testing already differentiates these disorders, miRNA profiling offers valuable insights into shared and distinct pathomechanisms underlying muscle involvement. This approach has particular value for understanding disease mechanisms and identifying potential therapeutic targets across neuromuscular conditions with overlapping clinical manifestations of muscle atrophy and weakness. Thus, miRNAs serve as molecular fingerprints of neuromuscular disorders, potentially complementing existing diagnostic approaches while providing deeper insights into shared pathological mechanisms.

## Single miRNAs as biomarkers and therapeutic targets: miR-23a and miR-34 in SMA

MiR-23a belongs to the miR-23/27/24 cluster, is highly expressed in neural tissues, and regulates neuronal differentiation, axon growth, and synaptic plasticity through modulation of key target genes [97, 98].

The miR-34 family (miR-34a, miR-34b, and miR-34c) contributes significantly to neuromuscular development and disease pathogenesis. MiR-34a specifically modulates neural stem cell proliferation and differentiation [99], regulates neurite growth and spinal morphology [100], and influences NMJ development, axon integrity, and endplate formation [101].

### Measurement strategies

Absolute quantification by droplet digital PCR is a reliable method for measuring low-abundance miRNAs, such as miR-23a, in biofluids (e.g., CSF) [102]. In addition, next-generation sequencing enables global expression profiling in multiple tissues and biological conditions [103]. However, the gold standard for quantifying miR-34 expression remains real-time RT-PCR [104], which has excellent sensitivity and specificity at very low concentrations, though with persistent difficulties in laboratory-to-laboratory standardization.

### Relevance to SMA

MiR-23a expression is severely dysregulated in SMA and reported to be downregulated in spinal cord tissue and motor neurons from SMA models; therefore, it is implicated in motor neuron loss [98, 105]. Mechanistically, miR-23a has a regulatory function in apoptotic signaling in motor neurons and has a potential regulatory function in neuron survival by virtue of indirect regulatory networks [106].

MiR-34 members decrease progressively in SMA patients treated with nusinersen [101]. Animal models have shown that miR-34 deficiency leads to SMA-like pathogenesis with ballooning of axons and loss of NMJ endplates, highlighting the relevance to motor neuron well-being and NMJ stability [101].

### Evidence from animal studies

Kaifer et al. [98] demonstrated significant miR-23a downregulation contributing to neuromuscular pathology in mouse models of SMA. Reintroduction via scAAV9 in the *SMN2*B/− model significantly improved disease outcomes, increasing motor neuron soma size and perimeter, improving NMJ integrity, enhancing the muscle fiber cross-sectional area, and extending survival [98]. This represents one of the first successful miRNA-based therapeutic interventions for SMA that does not directly target SMN protein.

Mice lacking the miR-34 family exhibit SMA-like pathology, particularly affecting axons and NMJ endplates. Therapeutic delivery of miR-34a using scAAV9 enhanced motor performance in SMN-Δ7 mice, correlating with restored NMJ endplate size [101].

ScAAV9-miR-23a treatment significantly reduced the disease severity in mouse models of SMA, improving multiple pathological aspects [98]. Similarly, AAV9-mediated miR-34a delivery had positive effects on motor function in animal models [101].

### Clinical studies

In vitro studies using motor neurons derived from SMA patient-derived induced pluripotent stem cells (iPSCs) showed approximately 2.5-fold downregulation of miR-23a expression compared to controls. Transfection of synthetic miR-23a rescued the neurons from cell death upon exposure to SMA astrocyte-conditioned media, revealing neuroprotective roles in human cells [98].

Chen et al. [101] analyzed CSF samples from seven SMA type I patients who were on nusinersen treatment. All three members of the miR-34 family had a tendency to decline 64 days after treatment compared to baseline, parallel with changes in phosphorylated neurofilament heavy protein (pNfH). Importantly, baseline CSF levels of miR-34b highly positively correlated (*r* = 0.79) with patient motor skills assessed by HINE-2 scores on day 482. This correlation withstood corrections for multiple comparisons, whereas the pNfH correlation did not, suggesting that levels of miR-34b at the start of treatment have the potential to predict the long-term treatment response [101].

### Function in diagnosis, prediction, and monitoring

MiR-23a has potential as a diagnostic biomarker by virtue of its 2.5-fold decrease in SMA motor neurons compared with controls [98]. MiR-34 also exhibits diagnostic potential due to its differential expression in SMA patients compared with healthy controls [101].

Animal studies suggest that miR-23a levels correlate with disease severity, and downregulation is associated with motor neuron pathology and muscle atrophy severity [98]. Baseline CSF miR-34 levels demonstrate significant predictive value for treatment outcomes, particularly when combined with other biomarkers, such as pNfH [101].

Preclinical models illustrate that modification of miR-23a expression is linked with motor neuron survival and improved NMJ integrity [98]. CSF miR-34 levels consistently present treatment-induced changes during nusinersen therapy, which can potentially provide information regarding the therapeutic response.

### Challenges and future directions

MiR-23a has limitations, including most available data being preclinical data, an incomplete understanding of protective mechanisms, unresolved questions about delivery methodologies, potential off-target effects, and an unknown correlation with SMN protein [98]. For miR-34, challenges include small clinical sample sizes, inconsistent normalization methods, lack of standardized collection protocols, difficulty identifying the most predictive family member, and limited understanding of SMN-miR-34 interactions. The contradictory findings between the therapeutic benefits of miR-34a in mice and its decreasing levels during successful human treatment require resolution.

Future research must tackle clinical validation in large cohorts with extended follow-up, standardization of quantification protocols across biofluids, testing of combinatorial therapy with SMN-targeting treatments, and delivery optimization. Multivariate panels of biomarkers that incorporate these miRNAs with known markers would improve the predictive capability. Basic mechanistic research must clarify the molecular mechanisms through which these miRNAs control motor neuron viability and muscle maintenance and consider potential cross-talk with SMN-dependent mechanisms [98, 101]. Longitudinal studies of miRNA expression during disease progression and the treatment response will determine their clinical utility as both biomarkers and targets for treatment.

## Other non-coding RNAs

NcRNAs are RNA molecules that are transcribed from DNA but not translated into proteins and that play critical regulatory roles in gene expression. Several ncRNA types show potential as SMA biomarkers, including Survival Motor Neuron Antisense Transcript 1 (SMN-AS1), an antisense lncRNA that negatively regulates the expression of SMN-encoding genes by recruiting epigenetic silencing complexes to the promoter; Metastasis Associated Lung Adenocarcinoma Transcript 1 (MALAT1), a conserved lncRNA regulating alternative splicing and nuclear organization that exhibits dysregulation in SMA; SINE element-containing RNA Upregulation (SINEUP), a synthetic antisense lncRNA that stimulates target mRNA translation without altering coding sequences, which is potentially useful for increasing SMN protein levels; and circular RNAs (circRNAs), which form closed-loop structures, with SMN circ4-2b-3 recently identified in exosomes from SMA cell lines and patient serum [107]. SMN circ4-2b-3 may identify nusinersen super-responders [107], with consistent expression maintained over time in both serum and CSF, though at lower levels in the latter. Additional ncRNAs with biomarker potential include small nuclear RNAs (snRNAs), small nucleolar RNAs (snoRNAs), and enhancer RNAs (eRNAs) [12, 25, 108–112]. CircRNAs may indicate disease severity, whereas snRNA and snoRNA modifications could track disease progression.

### Measurement strategies

Detection of ncRNAs relies on high-throughput sequencing technologies for both discovery and profiling in biological fluids. Careful sample handling is critical because freezing and repeated cycles of thawing followed by centrifugation can alter ncRNA patterns and compromise measurement accuracy. Handling protocols must be standardized for reproducible ncRNA biomarker research, particularly sample collection, storage, and handling [81, 113].

Exosomal ncRNAs from blood or CSF provide minimally invasive diagnostic alternatives to traditional testing methods. NcRNA levels demonstrate measurable changes during SMA treatment, enabling assessment of molecular therapeutic effects [109–111, 114].

### Relevance to SMA

NcRNAs contribute to SMA pathogenesis through multifaceted mechanisms. The antisense transcript SMN-AS1 suppresses transcription of genes encoding SMN through recruitment of polycomb repressive complex 2 to the promoter and is a novel therapeutic target for SMA [25, 108–111]. Targeted SMN-AS1 knockdown via antisense oligonucleotides increases SMN protein expression in murine central nervous system tissues, and patient-derived cell studies show that SMN-AS1 knockdown increases SMN protein expression, suggesting therapeutic potential.

SINEUP lncRNAs have potential to enhance the expression of full-length SMN transcripts, though specific SMN-targeting lncRNAs have yet to be identified. SnRNAs and snoRNAs contribute to RNA processing directly related to the fundamental function of SMN protein in snRNP assembly, which is essential in pre-mRNA splicing. MALAT1 is severely dysregulated in SMA models and affects neuron function, correlating with disease severity and progression. SMN-derived circRNAs contribute to transcriptional activity, and enhancer RNAs contribute to gene expression through RNA processing and splicing mechanisms [25, 109].

*SMN1/2* gene-derived circRNAs have been identified in patient samples as potential molecular signatures for disease monitoring. Guerra et al. [107] examined SMN circRNAs in 19 SMA type I patients treated with nusinersen, detecting SMN circ4-2b-3 in exosomes from both cell lines and patient serum. High copy numbers occurred in a subset of patients with superior treatment responses, suggesting its potential as a predictive biomarker. These findings require confirmation in larger cohorts, as not all patients with good responses to treatment have elevated levels of this circRNA [25, 109].

These findings support the role of ncRNAs in disease mechanisms, though their specific utility as biomarkers requires further investigation in pre-clinical models [25, 108].

### Challenges and future directions

Methodological inconsistencies in sample processing affect reliability across studies. Lack of standardized analytical protocols impairs reproducibility between research centers. Tissue-specific expression patterns complicate non-invasive monitoring, as ncRNA levels in accessible biofluids may not accurately reflect changes in the central nervous system or muscle tissue [81].

Future development of ncRNA biomarkers will require validation in representative patient cohorts, the establishment of standardized methods, and integration of ncRNA markers with other clinical markers. The most effective biomarker panels may be identified by machine learning, and therapeutic application of SMN-AS1 and SINEUP is promising. In the context of circRNAs, more research is required to confirm SMN circ4-2b-3 as a predictor as per Guerra et al. [107]. CircRNA exosome sorting may be used both as a biomarker and a therapeutic target [113].

## Coding RNA biomarkers in SMA

Coding RNAs (i.e., mRNAs) provide valuable insights into gene expression patterns and reflect the functional state of cells and tissues affected by SMA. Key coding RNA biomarkers include the mRNAs encoding SMN or Plastin 3 (PLS3); protective modifiers, such as coronin-1C (CORO1C), neurocalcin delta (NCALD), and calcineurin-like EF-hand protein 1 (CHP1); neuroprotective factors, including brain-derived neurotrophic factor (BDNF) and neurotrophin-3 (NT-3); and muscle-specific transcripts, such as MyoD, myogenin, and creatine kinase (CKM) [37, 46, 70, 115–117].

### Measurement strategies

Quantification of coding RNAs relies on well-documented molecular biology approaches. Real-time quantitative PCR is employed as a regular technique to analyze specific mRNA levels in blood samples. Muscle-specific markers traditionally require muscle biopsy samples, though recent advances have provided a means to identify a few of these in blood. RNA-seq provides global analysis of a number of coding RNAs at a given point in time and is capable of recognizing new patterns of expression.

### Relevance to SMA

Coding RNAs are central to SMA pathogenesis via diverse mechanisms. SMA animal models exhibit alterations in neuroprotective factors, with increased BDNF and NT-3 expression in transgenic mouse brains [115, 116]. These changes likely represent compensatory responses to motor neuron degeneration, suggesting potential therapeutic targets. Other protective modifiers (CORO1C, NCALD, and CHP1) are involved in essential cellular functions, such as calcium signaling, actin dynamics, and endocytosis, processes that are usually impaired in SMA [37, 70].

PLS3 is an actin-bundling protein that functions as a protective modifier, with overexpression being associated with milder phenotypes in female patients [70]. PLS3 expression correlates with disease severity, but specifically in post-pubertal female patients, where it is related to SMA type, gross motor function, and *SMN2* copy number. Strathmann [118] demonstrated that *PLS3* undergoes multilevel epigenetic regulation and escapes X-inactivation in a tissue-specific manner, explaining previously observed sex-specific protective effects and suggesting potential epigenetic therapeutic targets. The sex difference highlights complex interactions between hormonal regulation and modifier genes.

Muscle-coding RNAs exhibit typical muscle atrophy, and neuroprotective factors reveal underlying mechanisms of disease [115, 116]. Nicole et al. [119] revealed abnormal expression of muscle growth regulators in SMA muscle biopsy samples, with altered patterns of MyoD, myogenin, and other muscle-specific transcripts corresponding to the severity of muscle atrophy [117, 119]. Longitudinal investigations of SMN transcript levels in response to SMN-enhancing therapies have provided valuable pharmacodynamic insights.

### Functions of the biomarkers

PLS3 levels show potential as predictive markers for diagnosis and prognosis, particularly in female patients. *PLS3* expression in blood cells can serve as a prognostic indicator in specific patient subgroups, predicting disease course and potentially guiding treatment decisions [70].

Muscle-specific RNAs (MyoD, myogenin, CKM) in muscle biopsies provide markers for longitudinally tracking muscle pathology. These transcripts reflect changes in muscle development and regeneration processes disrupted in SMA. Recent research has focused on identifying circulating markers as less invasive alternatives to muscle biopsies.

### Challenges and future directions

The clinical application of coding RNA biomarkers faces significant challenges. The age- and sex-specific effects of PLS3 (i.e., correlation with disease severity only in post-pubertal females) limits its broad applicability across the SMA population, suggesting hormonal influences that require further investigation. Technical challenges include RNA stability in clinical samples and the invasive nature of muscle biopsies for measuring muscle-specific transcripts, which is particularly problematic for pediatric monitoring.

Future research must include validation in large cohorts and the development of standardized clinical analysis protocols. Integration of coding RNA biomarkers with protein measurements and ncRNAs would make more comprehensive panels with more diagnostic utility. More sensitive technologies, such as droplet digital PCR and targeted RNA-seq, would allow longitudinal monitoring with decreased invasiveness. Combined panels of coding RNA markers would improve the understanding of progression and the response to treatment and facilitate more personalized management of SMA.

## Multiple RNAs and panel analysis in SMA: towards comprehensive biomarker profiling

Multiple-RNA panel analysis represents a comprehensive approach to SMA biomarker profiling that simultaneously examines various RNA species, including cell-free RNA (cfRNA), exosomal mRNAs, and alternatively spliced transcripts. This methodology captures the complex molecular landscape of SMA by analyzing multiple markers together rather than individual transcripts in isolation [120–122]. The panels incorporate both coding RNAs (i.e., mRNAs) and ncRNAs, providing a complete view of disease-related transcriptional changes [123].

### Measurement strategies

Advanced technologies employed for multiple-RNA analysis include high-throughput RNA-seq, by which in-depth transcriptome analysis with unbiased profiling of thousands of transcripts is achieved at once [124]. The nCounter NanoString technique analyzes transcriptional changes in tissue samples and blood cells, delivering sensitivity and reproducibility with no bias in amplification. Exosomal mRNA analysis requires specific vesicle isolation and RNA extraction protocols, and analysis of cfRNA in CSF requires specific protocols with strict quality control [125, 126]. Analysis of muscle-specific transcripts (e.g., MyoD, myogenin, CKM) typically requires biopsy samples [127].

### Relevance to SMA

Multiple-RNA analysis identifies complex changes in gene expression patterns in RNA processing pathways, synaptic function, and axonal transport. Splicing patterns that are dependent on the SMN protein level introduce additional information that is relevant to disease. The technique captures both SMN-independent and SMN-dependent molecular changes and provides a more complete picture of disease mechanisms [120, 121]. The fine-grained profiles identify both indicators of the disease state and candidate therapeutic targets outside of SMN that can complement existing treatments.

Clinical research analyzing cfRNA in CSF from pediatric SMA type II and III patients after nusinersen treatment has identified 48 differentially expressed cfRNAs, including disease-specific genes (*ARHGEF19*, *COX6C*, *MTMR14*, *KCNK2*) and treatment-specific genes (*HNRNPA1L2*, *SAP18*, *DDX19B*). Leo et al. [126] found alterations in four genes (*AMIGO1*, *CA2*, *CCL5*, *TLR2*) that remained unaffected by nusinersen treatment in late-onset SMA patients [125, 126]. Studies integrating multiple RNA types have shown that certain miRNAs regulate key transcripts involved in SMA pathogenesis, providing multi-level insight into affected regulatory networks [128].

### Function in monitoring disease progression and therapeutic response

Multiple-RNA panels combined with genetic and clinical parameters improve patient stratification in diagnosis and prognosis. Transcriptional signatures can potentially distinguish between SMA subtypes and predict disease progression more accurately than *SMN2* copy number alone [122]. Exosomal mRNAs and cfRNAs offer minimally invasive approaches for monitoring molecular changes over time. RNA profile changes may precede clinical changes, providing early indicators of disease progression [56].

This approach distinguishes between disease-specific and treatment-specific markers, as demonstrated in nusinersen-treated patients. This distinction helps determine whether treatments address core disease mechanisms or merely compensatory pathways [125]. Panels also identify potential targets for complementary therapies, particularly for patients with a limited response to SMN-enhancing treatments [129].

### Challenges and future directions

Patient subgroups with varying cfRNA expression profiles lack a clear correlation with clinical phenotypes or treatment responses, creating challenges in interpretation and highlighting the gap between molecular changes and clinical outcomes [123]. Technical barriers include protocol standardization across laboratories and the need for advanced bioinformatics to extract meaningful information from complex RNA data [130]. The discovery of SMN-independent transcriptional changes reveals limitations in SMN-focused therapies, suggesting the need for complementary approaches targeting alternative pathways.

Future research needs to integrate RNA panels with other molecular and clinical markers and machine learning to identify the most efficient biomarker panels for improved diagnostics [130]. Profiling panels require validation in heterogeneous cohorts, and less invasive methods for muscle-specific transcript analysis would be advantageous in clinical monitoring. Multi-omic integration with proteomic, metabolomic, and clinical data [129] has potential for more personalized treatment in patients with a poor response to SMN-augmenting therapy.

## Transcriptome-wide changes in SMA: expanding the biomarker landscape

Transcriptome-wide changes in SMA represent comprehensive alterations in gene expression patterns extending beyond SMN. High-throughput sequencing technologies have revealed complex dysregulation of gene expression throughout the genome. This approach encompasses global gene expression changes, alternative splicing patterns, ncRNA expression, and gene expression networks, analyzing both protein-coding and non-coding genes to provide a broad view of transcriptional changes in SMA [82, 131–133]. These transcriptomic alterations advance understanding of disease mechanisms and provide potential biomarkers for diagnosis, prognosis, and treatment monitoring.

### Measurement strategies

A range of advanced technologies are employed in transcriptome analysis. High-throughput RNA-seq has been the principal technique employed in global gene expression analysis in SMA models and patient samples [134, 135]. Single-cell RNA-seq enables cellular-scale analysis and measurement of cell type-specific changes in gene expression in the spinal cord and the discovery of unique transcriptional signatures in motor neurons, astrocytes, and other cell types [135–138]. Weighted gene co-expression network analysis identifies modules of coordinately regulated genes. Other methods are patient-derived organoids, iPSC models, liquid biopsy approaches, and machine learning tools for advanced data analysis [139].

### Relevance to SMA

Transcriptome-scale research has made significant contributions to SMA pathogenesis through a variety of mechanisms. RNA-seq has revealed global changes in gene expression in tissues, identifying dysregulated genes involved in muscle growth, energy metabolism, neural development, synaptic function, RNA splicing, and apoptosis [117, 131, 135, 140]. Alternative splicing in U12-introns and calcium channel genes are particularly important in understanding disease mechanisms [132, 135]. Multiple differentially expressed RNAs have been found in SMA patient-derived motor neurons [82, 136, 141], and specific markers (e.g., NRXN2 and SYNCRIP) have been found to be potential therapeutic targets. Consistently dysregulated gene expression modules in weighted gene co-expression network analysis have been found to be those involved in synapse maintenance and formation.

### Evidence from animal studies

SMA mouse models have revealed significant transcriptional changes. Zhang et al. [131] identified approximately 300 differentially expressed genes in spinal cord motor neurons and white matter on presymptomatic PND1, including genes critical for neuron development, synaptic function, and RNA processing. They discovered complete skipping of agrin Z exons essential for NMJ maintenance, strong upregulation of complement factor C1q involved in synapse pruning, and downregulation of the Etv1/ER81 transcription factor required for sensory-motor circuit formation. These transcriptome abnormalities precede motor neuron pathology, providing a molecular link between SMN deficiency and the characteristic synaptic defects of SMA [131].

Bricceno et al. [117] reported altered expression of genes involved in pathways related to muscle growth, energy metabolism, and cell death in muscles from a mouse model of SMA [117]. SMN-deficient muscle cells had altered expression of myogenic regulatory factors, with premature MyoD and myogenin upregulation in proliferating cells but reduced induction during differentiation.

Doktor et al. [28] demonstrated widespread splicing alterations, particularly in U12-introns, in tissues from SMA mice, which improved with antisense oligonucleotide treatment [28]. Their comprehensive RNA-seq analysis revealed incorrect splicing in calcium channel genes (*Cacna1a, Cacna1b, Cacna1c, Cacna1e, Cacna1h*), potentially explaining the altered calcium homeostasis in SMA cells. The study confirmed that aberrant U12-intron retention occurs early after the reduction in SMN levels and affects tissues beyond the central nervous system [28].

### Clinical studies

Rizzo et al. [136] performed RNA-seq on iPSC-derived motor neurons from SMA patients, revealing 1,084 downregulated and 808 upregulated genes compared to controls. Their analysis identified significant reductions in transcripts related to axon-related proteins (STMN2, PLP1), potassium channels, and synaptic proteins (SYT13, neurexins 1–3). Through differential splicing analysis, they detected 12,144 deregulated cassette exons. Motif enrichment analysis identified a common sequence motif recognized by SYNCRIP, an SMN-interacting RNA-binding protein [136].

Grass et al. [137] used isogenic patient-derived organoids to uncover neurodevelopmental defects in SMA. Their longitudinal gene expression analysis revealed alterations in neural lineage specification, including reduced expression of Neural Stem Cell markers SOX2 and NESTIN and downregulation of pro-neural gene *NGN2*. Temporal transcriptomic profiling showed aberrant expression of neuronal markers (DCX, SMI-32) and motor neuron-specific genes (*NKX6.1*, *HB9, ISL1, CHAT*), with premature upregulation of terminal differentiation markers, suggesting accelerated but defective neuronal specification. Single-cell RNA-seq identified bias toward mesodermal lineage commitment at the expense of the neuroectodermal fate. Notably, these gene expression abnormalities were only partially corrected by an *SMN2*-to-*SMN1* conversion, suggesting that early developmental transcriptional dysregulation may contribute to pathology despite increased levels of SMN protein [137].

### Biomarker utility

Kumar et al. [135] performed a meta-analysis of transcriptomics studies in SMA across different tissues and mouse models. They identified few consistently dysregulated genes across tissues, with Metallothionein 2 (*Mt2*) among the common differentially expressed genes involved in oxidative stress and detoxification. Many genes and pathways appear to play tissue-specific roles in SMA, with contractile fibers and myosin complexes being significant Gene Ontology terms across multiple comparisons [135].

The transcriptome-wide approach serves multiple functions in SMA management. For diagnosis and prognosis, it provides complex biomarker signatures that can predict disease severity and progression, particularly through machine learning approaches [139]. For disease monitoring, it offers a comprehensive assessment of molecular changes across tissues and cell types [117]. This approach also tracks global changes following therapeutic interventions, as demonstrated in antisense oligonucleotides treatment studies where splicing errors were reduced and gene changes reversed [141]. The approach identifies potential therapeutic targets beyond SMN-targeting approaches.

### Challenges and future directions

Translating complex transcriptome changes into clinically useful biomarkers, standardizing assays, and integrating multiple data types are key challenges in future research. The persistence of transcriptome changes even after an *SMN2*-to-*SMN1* conversion suggests limitations in current therapeutic approaches. Technical challenges include RNA stability in clinical samples and complex data analysis requirements.

Future studies should integrate RNA-seq, single-cell RNA-seq, and multi-omics analyses for a comprehensive view of molecular changes across tissues and cell types [138, 142]. Research priorities include validating biomarker candidates in larger patient cohorts, developing standardized assays, and integrating them with existing clinical and molecular markers [139]. Machine learning algorithms and multi-omics approaches show promise for developing personalized prognostic tools, whereas liquid biopsy techniques could enable longitudinal monitoring without repeated tissue biopsies. As technologies advance and understanding of the molecular landscape of SMA deepens, these approaches may enable more precise and personalized management strategies.

## Future directions in SMA biomarker research

No biomarker yet fulfills all clinical functions (i.e., diagnosis, prediction, prognosis, monitoring, therapeutic response, and pharmacokinetics) in SMA. Combined approaches offer the most promising future directions. Protein/RNA panels, SMA scores, and untargeted -omics methodologies, along with whole miRNome and transcriptome analyses, have revealed unique molecular signatures in SMA. Integrating these approaches with clinical data could yield comprehensive biomarker panels for accurate diagnosis, prognosis, and treatment monitoring [143]. Furthermore, combining molecular and non-molecular biomarkers, such as MRI and neurophysiological measures, has significant potential for improving SMA management [76, 121, 122].

Peripheral biomarkers have gained renewed interest, with breakthroughs in circulating neurofilament research suggesting that blood sampling could provide meaningful insights, especially in infant SMA cohorts [19, 20, 144]. In addition, ncRNAs (miRNAs, lncRNAs, circRNAs) deserve attention as biomarkers due to their stability, tissue specificity, and amplificability [112]. Plasma miR-9, miR-132, and miR-206 are also promising minimally invasive biomarkers [24, 62]. However, CSF remains highly promising as a source of biomarkers due to its proximity to affected areas, but standardized procedures for sample handling, storage, and analysis are essential. Leveraging CSF collected during therapeutic procedures, such as intrathecal nusinersen injections, offers unique opportunities for biomarker exploration [20, 40, 144].

With advances in SMA treatments, such as antisense oligonucleotides [24], gene therapy [28, 116], and small molecules [145], RNA biomarkers could play critical roles in monitoring treatment efficacy. Changes in specific ncRNA levels after treatment could be employed as pharmacodynamic markers [45]. Predictive modeling with molecular biomarkers and clinical parameters, potentially supported by artificial intelligence, could enable more personalized therapeutic decisions [129, 139]. With greater prevalence of newborn screening, rational biomarker research is increasingly needed because early intervention is not always successful [5, 8, 12]. Baseline biomarker samples are essential for designing efficient research plans at early stages of biomarker implementation. Careful experimental design can significantly contribute to the long-term success of SMA biomarkers and potentially improve our understanding of SMA and other neuromuscular diseases [143].

SMA biomarker research has advanced significantly over three decades, with gene-targeted treatments opening new avenues for biomarker development [5]. Though substantial progress has been made, especially with RNA-based markers [146], validation in larger patient cohorts and standardized measurement protocols remain essential. Combining RNA biomarkers with other molecular indicators and clinical information could lead to more comprehensive management approaches for SMA, ultimately enabling more effective prediction, diagnosis, and treatment [147–150].

## Data Availability

Not applicable.
